# Are Nigerian Women Pro-Active about Noncommunicable Disease Prevention? A Quantitative Survey

**DOI:** 10.5334/aogh.2355

**Published:** 2019-03-15

**Authors:** Oluwatoyin Aribike, Ifeoma Okafor, Alero Roberts, Tinuola Odugbemi

**Affiliations:** 1 College of Medicine, University of Lagos, NG

## Abstract

**Background:**

Noncommunicable diseases (NCDs) continue to cause significant morbidity and mortality worldwide with incidence increasing rapidly in developing countries. Poor utilization of preventive healthcare services contributes to this high burden.

**Objective:**

To assess the knowledge and utilization of preventive healthcare services among women in Lagos, Nigeria.

**Methods:**

This was a cross-sectional descriptive study carried out at Mainland Local Government Area (LGA) between May and July 2014. Respondents were selected using the multistage sampling method. A pretested, interviewer-administered questionnaire was used to obtain information. Data were analyzed using Epi info software version 7. Summary and inferential statistics were done and the level of significance was set at <5% (p < 0.05).

**Findings:**

Awareness of specified NCDs among the 322 respondents was 82.61% and of preventive healthcare services for the NCDs was 65.22%. Virtually all (99.05%) of the respondents had poor knowledge of these preventive services. Utilization rates were equally poor. Most common screening/tests done were Blood Pressure measurement (78.18%), Self breast examination (69.96%) and blood sugar test (58.33%). Much lower utilization rates were recorded for lipid profile (37.57%), Pap smear (26.11%), Visual Inspection with Acetic Acid (VIA) (19.72%), Human Papilloma Virus (HPV) immunization (16.55%) and mammography (14.72%).

**Conclusions:**

Respondents were aware of specified NCDs and preventive healthcare services. They considered routine medical check-up important, however they had poor knowledge of the preventive health services for NCDs and hardly utilized them. Women should be given detailed information on the preventive healthcare services to improve their knowledge and utilization so as to reduce the NCD burden.

## Introduction

Over the years, the incidence of chronic diseases progressively increased till it became the major cause of morbidity and mortality in the world [1]. This increase is affecting both developing and developed countries alike and can be attributed to similar lifestyle practices.

Most Noncommunicable diseases (NCDs) tend to have similar risk factors including tobacco use, physical inactivity, unhealthy diet and lack of utilization of preventive healthcare facilities [1].

NCDs are now the leading cause of mortality in the world (about 60%) [1] with half of the mortalities occurring in women [2]. An estimated 80% of these deaths occurred in lower income populations, 29% occurring before the age of 60 years [1]. NCD mortality rate is about 90% in high income countries, 30% in Africa and 28% in Nigeria [3]. Common NCDs encountered in Nigeria include: Cardiovascular diseases (CVDs) with 12% mortality rate, cancers and non-communicable variants of respiratory diseases with 3% mortality rate, diabetes mellitus (DM) with 2% mortality rate and other NCDs accounting for 7% of the mortalities [4].

Projections indicate that these figures will rise in the coming decades [5], especially in Africa where NCD morbidity and mortality is predicted to show an increase from 28 to 60% and 35 to 65% respectively [6] with the increase occurring more in women [7]. Contributing to the rising incidence and prevalence of NCDs in low- and middle-income countries (LMICs) are several factors such as; lifestyles, poverty, uneven distribution of wealth, lack of education and rapid urbanization. Other factors include the economic social, gender, political, behavioural and environmental determinants of health [8].

The burden of NCDs is higher in LMICs partly because most of the preventive/curative healthcare services are paid for out-of-pocket, leading to strain on the affected households. The costs to individuals, businesses, governments and healthcare systems add up to major macroeconomic impacts. Economic analysts also suggest that each 10% rise in chronic diseases is associated with 0.5% lower rates of annual economic growth [1].

More than half of the NCD burden could be avoided and majority of premature deaths prevented through health promotion and prevention initiatives [9,10]. This highlights the need for more emphasis on preventive healthcare services, the goal of which is to protect, promote and maintain health and well-being to prevent morbidity and mortality [11]. Clinical preventive services for NCDs at the individual level include immunizations, screening tests for early detection, counseling for health behavior-related change and chemoprevention [12]. According to the World Health Organization (WHO), population-wide primary prevention and individual healthcare methods have contributed to the decline in cardiovascular disease related deaths [1]. Improvements noted in the outcome of cancer patients in high income countries have also been attributed to early detection and greater access to care and screening interventions [11]. In line with global health goals, countries need to work towards achieving the ambitious target of a 25% reduction of premature mortality from the four major NCDs by 2025 [9].

Cost effective preventive services including screening for high blood pressure, high blood sugar, obesity and tests such as visual inspection with acetic acid (VIA), pap smear as well as screening for eye, dental, oral and mental abnormalities should be utilized as a means of reducing the burden from chronic diseases [8,13].

This study is focused on women in their most productive years. NCDs are said to be the biggest threat to women’s health worldwide with increasing impact on women in developing countries during their productive years [2]. They are also less likely to be diagnosed with NCDs at an early stage [7]. While bearing a disproportionate share of the burden of care-giving in the family, they tend to pay little or no attention to their own health. Female mortality as a result of NCDs has several negative consequences on the family and community as a whole. It tends to lead to increased mortality among younger children, food insecurity, increased incidence of children withdrawing from school and child labour to make ends meet [2]. Even when they don’t have the disease, women are indirectly affected by NCDs. As the primary caregivers in the home, they are often obliged to stay home to care for the sick family member thereby interrupting their education or income earning abilities [2].

Furthermore, maternal and child health are indistinguishably linked with NCDs and their risk factors in various ways. Prenatal malnutrition and low birth weights predispose to obesity, cardiovascular diseases and DM [2,3,4,5,6,7,8] while obstetric conditions such as obesity and gestational diabetes are associated with similar risks in both mother and child [8].

At the 66th session of the UN General Assembly (UNGA), member states resolved to pursue and promote gender-based approaches for the prevention and control of NCDs to address the critical differences in the risks of morbidity and mortality from NCDs for women and men [14,15]. Partnering with women can help the fight against NCDs as women have been said to be potentially instrumental in decreasing the modifiable risk factors from the household and even beyond [2].

Based on the significance of NCDs as a growing global public health problem and the gender roles in disease risk, this study was conducted to assess the awareness and utilization of preventive healthcare services among women in Lagos State, Southwest Nigeria. Its objectives include knowledge, utilization and factors affecting the knowledge and utilization of preventive healthcare services. Findings will be useful in the planning and implementation of intervention programmes to reduce the NCD burden in addition to scaling of the existing ones.

## Methods

### Background to study area

Nigeria is a heavily populated country with limited health resources. Over the years, the Federal Ministry of Health (FMoH) has made efforts to reduce the burden of NCDs in Nigeria. The NCD Control Programme is now a division in the FMoH and a National Strategic Plan on NCDs has also been recently published [16]. However, attempts to fully integrate NCDs prevention and control into the primary healthcare system have been made with limited success.

Lagos is one of the most populated states in Nigeria with a projected population of almost 20 million. Administratively, it has 20 Local Government Areas (LGAs) which are further organized into wards [17]. Lagos State conducts a hypertension and diabetes awareness and screening program which is a coordinated state-wide exercise. The program has now been incorporated into an integrated health screening package referred to as the ‘Lagos State-wide Wellness Week Screening programme.’ This health screening programme involves creating awareness and screening for hypertension and diabetes as well as screenings for cancers of the breast, cervix and prostate. There are three state-owned mammography machines and a few privately-owned ones.

Lagos Mainland is one of the 20 LGAs in the state and has a total population of 629,469 with 326,433 males and 303,036 females [17]. It has 18 political wards. Most inhabitants are lower-middle-class. The women are mainly petty traders, civil servants and housewives. At the time of the study, there were a total of 88 registered health facilities (13 public and 75 private facilities) in the area. Alternative healthcare providers also operate in the area. None of the public health facilities where mammography is available is located in Mainland LGA. For residents, payment for preventive healthcare services are mainly out-of-pocket with the exception of the few who have Health Insurance packages or the periodic screening exercises carried out by the government, Non-Governmental Organizations (NGOs), religious institutions and corporate bodies.

### Study design and population

This was a descriptive, cross-sectional, community-based study. Only females aged 18–65 years who had been residents of Mainland LGA for at least one year were included.

### Sample size

The required sample size was determined to be 293 respondents using the formula for descriptive studies: n = Z^2^pq/e2 where Z = standard normal deviate at 95% confidence interval (1.96), p = 74.4% (Proportion of women who had done a blood sugar test) [18] and margin of error (0.05). To accommodate non-responses, 10% of the minimum sample size calculated above was added, and a total of 322 respondents were interviewed. All of them responded appropriately and the questionnaires analyzed.

### Sampling methodology

Multistage sampling method was used to select the respondents.

**Stage one (selection of wards):** Mainland LGA has a total of 18 political wards. Three of the wards- Alobadesalu, Oko-baba and Otto were selected by simple random sampling (balloting). Equal proportions of respondents were allocated to each ward thus, a total of 107 respondents were required from each ward.

**Stage two (selection of streets):** There were between 20–30 streets in each ward and 10 streets were selected by simple random sampling (balloting). About 11 respondents were required from each street.

**Stage three (selection of houses):** There were approximately 40 houses in each street and 11 were selected by the simple random sampling method (balloting).

**Stage four (selection of respondents):** An adult female between 18 and 65 years was selected in each house. Where there was more than one female who met the inclusion criteria in the house, only one was selected by simple random sampling (balloting).

### Data collection and analysis

Data was collected in the second quarter of 2014 by four trained female interviewers using a pre-tested, structured, interviewer-administered questionnaire. Interviews were conducted in English, Yoruba (the indigenous language) or pidgin English (a local variant of the English Language). The interviewers were all fluent in these languages. Each interview lasted for about 20 minutes. The survey tool used for the study was developed by the researchers. Questions where informed by consultations with experts in the area of NCDs, relevant formal healthcare providers and fellow researchers.

Data entry and analysis were done using EPI Info Version 7. The quantitative data generated from the study were analyzed using summary statistics (proportions, mean) and inferential statistics (Chi square test). Level of significance was set at 5% (p < 0.05).

Awareness of specified diseases and preventive healthcare services were scored and graded to obtain summary measures. The diseases considered were Breast cancer, Cervical cancer, Dibetes Mellitus, High blood pressure, Hyperlipidaemia, Glaucoma, Refractive errors, Cataract, Dental Caries. One mark was awarded for each disease mentioned. The maximum score was 9 (100%). Scores <50% were graded as poor and ≥50% as good.

For preventive healthcare services, one mark was awarded for each service mentioned. The maximum score was 13 (100%). Scores <50% were graded as poor while ≥50% were good.

Respondents’ knowledge of preventive health services was assessed with the following parameters: corresponding disease and the preventive test/service; ideal age to commence utilization of service; frequency of utilization of the preventive service. As at the time of study, annual medical check-up was recommended in Nigeria, for most of the NCDs like DM, hypertension, breast cancer, hyperlipidaemia as well as eye and dental check-up, and mammogram for those above 40 years. Cervical cancer screening should be three yearly for sexually active women and HPV vaccination taken according to schedule. Knowledge was summarized by scoring and grading. One mark was awarded for each correct response and no mark was given for incorrect or non-responses. The maximum score was 33 (100%). Knowledge was graded as poor (<50%) or good (≥50%).

For utilization, respondents were asked whether they had ever utilized the following services: self-breast examination (SBE), clinical-breast examination (CBE), mammogram, Pap smear, visual inspection tests for cervical cancer: Visual Inspection with Acetic Acid (VIA) and Visual Inspection with Lugol’s iodine (VILI), Human Papilloma Virus (HPV) vaccination, blood sugar test (BST) ie random blood sugar, blood pressure (BP) measurement, lipid profile, body mass index (BMI) evaluation and waist circumference (WC) measurement. In addition, information on awareness and utilization of preventive dental and eye healthcare services were also collected.

### Ethical considerations

Ethical approval for this study was obtained from the Health Research and Ethics Committee of Lagos University Teaching Hospital (LUTH). Permission was also obtained from the Local Government Chairman of Lagos Mainland LGA through the Medical Officer of Health. Written informed consent was obtained from respondents prior to interview. They were informed of their right to withdraw from the study at any time. Anonymous questionnaires were used so as to maintain confidentiality.

## Results

### Socio-demographic characteristics

The ages ranged from 18 to 65 years. The mean age was 34.24 ± 13.54 years. Most (68.94%) of the respondents were Yoruba and Christian (77.33%), 63.98% of the respondents were educated beyond the secondary level. Most (57.45%) of them were either married/co-habiting. Majority (62.73%) were employed but 38.51% earned less than the minimum monthly income in Nigeria (18,000 Naira ~ 50 US Dollars) monthly (Table 1).

**Table 1:** Socio-demographic characteristics of respondents.

Variable	Frequency	Percentage

**Age (years)**		

<21	55	17.08%
21–30	112	34.78%
31–40	69	21.43%
41–50	34	10.56%
51–60	32	9.94%
>60	20	6.21%
**TOTAL**	**322**	**100%**
**MEAN ± S.D**	34.24 ± 13.54	
**Ethnicity**		

Yoruba	222	68.94%
Igbo	70	21.74%
Hausa	8	2.48%
Others	22	6.83%
**Total**	**322**	**100%**
**Religion**		

Christianity	249	77.33%
Islam	73	22.67%
**Total**	**322**	**100%**
**Level of education**		

None	23	7.14%
Primary	13	4.04%
Secondary	80	24.84%
Post-secondary	206	63.98%
**Total**	**322**	**100%**
**Marital status**		

Never married	121	37.58%
Married/co-habiting	185	57.45%
Widowed/separated/divorced	16	4.97%
**Total**	**322**	**100%**
**Employment status**		

Employed	202	62.73%
Unemployed	120	37.27%
**Total**	**322**	**100%**
**Estimated monthly income (Naira)**		

<18,000	124	38.51%
>18,000–<40,000	83	25.78%
>40,000–<60,000	38	11.80%
>60,000–<80,000	16	4.97%
>80,000–<100,000	13	4.04%
>100,000	48	14.91%
**Total**	**322**	**100%**

### Awareness of specified NCDs and preventive healthcare services/tests

Majority of the respondents were aware of the various diseases, 1.24% of the respondents were not aware of any of the specified diseases. The most common conditions known were high blood pressure (97.52%), breast cancer (95.03%) and diabetes mellitus (95.03%).

A majority (82.61%) of the respondents were aware of at least half of the specified diseases. The common NCD preventive healthcare services the women were aware of include: SBE 78.57%, CBE 72.36%, Blood Sugar testing 93.17% and BP measurement 95.34%. Only a few (22.05%) women were aware of VIA/VILI (Table 2).

**Table 2:** Awareness of specified diseases and preventive health services.

Disease (n = 322)	Freq (%)

**High blood pressure**	314 (97.52)
**Breast cancer**	306 (95.03)
**Diabetes mellitus**	306 (95.03)
**Refractive errors**	236 (73.29)
**Cervical cancer**	225 (69.88)
**Dental caries**	222 (68.94)
**Hyperlipidemia**	217 (67.39)
**Cataract**	212 (65.84)
**Glaucoma**	171 (53.11)
**Preventive health services/test (n = 322)**	**Freq (%)**

**Blood pressure measurement**	307 (95.34)
**Blood sugar test**	300 (93.17)
**Self-breast examination**	253 (78.57)
**Clinical breast examination**	233 (72.36)
**Lipid profile**	181 (56.21)
**Pap smear**	157 (48.76)
**BMI calculation**	152 (47.20)
**Mammogram**	142 (44.10)
**HPV vaccination**	139 (43.17)
**Waist circumference measurement**	119 (36.96)
**VIA/VILI**	71 (22.05)

* Multiple responses allowed.

### Sources of information on preventive healthcare services

Most common source of information was the healthcare worker for all the preventive services especially dental caries (74.32%) and hyperlipidemia (70.51%) and also for the preventive services especially lipid profile test (85.64%), waist circumference measurement (81.51%), blood sugar test (81.33%) and blood pressure measurement (81.10%). Other sources of information included electronic and print media, social media, family and place of work (Table 3).

**Table 3:** Sources of information on specified preventive health services.

Variable	Healthcare workers (%)	Electronic media (%)	Social media (%)	Print media (%)	Family (%)	Place of work (%)	Others (%)

**Self-breast examination (n = 253)**	196 (77.47)	100 (39.53)	56 (22.13)	56 (22.13)	42 (16.60)	27 (10.67)	27 (10.67)
**Clinical breast examination (n = 233)**	184 (78.97)	75 (29.64)	48 (20.60)	44 (18.88)	29 (12.44)	23 (9.87)	26 (11.16)
**Mammogram (n = 142)**	110 (77.46)	55 (38.73)	38 (26.76)	44 (30.99)	23 (16.20)	21 (14.79)	24 (16.90)
**Pap smear (n = 157)**	127 (80.89)	52 (33.12)	34 (21.66)	40 (25.48)	23 (14.65)	20 (12.74)	23 (14.65)
**HPV vaccination (n = 139)**	111 (79.86)	42 (30.21)	25 (17.99)	27 (19.42)	18 (12.95)	17 (12.23)	21 (15.11)
**VIA/VILI (n = 71)**	54 (76.06)	27 (38.03)	16 (22.54)	21 (29.58)	12 (16.90)	15 (21.13)	15 (21.13)
**Blood sugar test (n = 300)**	244 (81.33)	97 (32.33)	43 (14.33)	51 (17.00)	45 (15.00)	27 (9.00)	25 (8.33)
**Blood pressure test (n = 307)**	249 (81.10)	98 (31.92)	51 (16.61)	61 (19.87)	58 (18.87)	36 (11.73)	24 (7.82)
**Lipid profile (n = 181)**	155 (85.64)	62 (34.25)	30 (16.57)	41 (22.65)	26 (14.36)	27 (14.92)	24 (13.26)
**(BMI) evaluation (n = 152)**	119 (78.29)	41 (26.97)	27 (17.76)	31 (20.39)	23 (15.13)	23 (15.13)	21 (13.82)
**Waist circumference (n = 119)**	97 (81.51)	30 (25.21)	25 (21.01)	24 (20.17)	15 (12.61)	15 (12.61)	22 (18.49)

* Multiple responses allowed.

### Knowledge of NCD preventive healthcare services

More than half of the respondents answered correctly the disease for which the following preventive services were for: SBE (70.36%), CBE (67.38%), mammogram (58.45%), Pap smear (52.86%), BST (52.00%). Majority of the respondents did not know the correct age for the tests and standard frequency for each preventive service (Table 4).

**Table 4:** Knowledge on the specified preventive healthcare services.

Variable	Disease which the preventive service is diagnosing (%)	Ideal age to begin the preventive service (%)	Ideal frequency for the preventive service (%)

Self breast examination (n = 253)			

correct	178 (70.36)	55 (21.74)	41 (16. 21)
incorrect	75 (29.64)	198 (78.26)	212 (83.79)
**Clinical breast examination (n = 233)**			

correct	157 (67.38)	43 (18.46)	20 (8.58)
incorrect	76 (32.62)	190 (81.54)	213 (91.42)
**Mammogram (n = 142)**			

correct	83 (58.45)	15 (10.56)	4 (2.82)
incorrect	9 (41.55)	127 (89.44)	138 (97.18)
**Pap smear (n = 157)**			

correct	83 (52.87)	5 (3.19)	9 (5.73)
incorrect	74 (47.13)	152 (96.81)	148 (94.26)
**VIA/VILI (n = 71)**			

correct	13 (18.31)	2 (2.82)	2 (2.82)
incorrect	58 (81.69)	69 (97.18)	69 (97.18)
**Blood sugar test (n = 300)**			

correct	156 (52.00)	4 (1.33)	2 (0.67)
incorrect	144(48.00)	296 (98.67)	298 (99.33)
**Blood pressure test (n = 307)**			

correct	148 (48.21)	30 (9.77)	2 (0.65)
incorrect	159 (51.79)	277(90.23)	305 (99.35)
**Lipid profile (n = 181)**			

correct	86 (47.51)	14 (7.73)	1 (0.55)
incorrect	95 (52.49)	167 (92.27)	180 (99.45)
**BMI evaluation (n = 152)**			

correct	58 (38.16)	10 (6.58)	1 (0.66)
incorrect	94 (61.84)	142 (93.42)	151 (99.34)
**Waist circumference (n = 119)**			

correct	40 (33.61)	6 (5.04)	1 (0.84)
incorrect	79 (66.39)	113 (94.96)	118 (99.16)
**HPV vaccination (n = 139)**			

correct	51 (36.69)	12 (8.63)	12 (8.63)
incorrect	88 (63.31)	127 (91.34)	127 (91.34)

### Utilization of NCD preventive healthcare services

Majority of the respondents who knew about SBE (69.96%), blood sugar test (58.33%) and blood pressure measurement (78.18%) had done the tests before. The proportions of women who had done other tests were in the minority. The women who had done any of the tests at least once in the past year were very few except SBE. Majority (80.79%) of the women had done it at least once in the past year. Most common location for the tests was General Hospital except for VIA/VILI where the primary healthcare center (PHC) was the most common location. (Table 5).

**Table 5:** Utilization of preventive healthcare services.

Preventive healthcare services utilized by the respondents	Yes (%)	No (%)	Number of times in the past year	Frequency (%)

**Self breast examination (n = 253)**	177 (69.96)	76 (30.04)	<1	34 (19.21)
			≥1	143 (80.79)
**Clinical breast examination (n = 233)**	105 (45.06)	128 (54.94)	<1	52 (49.52)
			≥1	53 (50.48)
**Mammogram (n = 142)**	21 (14.79)	121 (85.21)	<1	11 (52.38)
			≥1	10 (47.62)
**VIA/VILI (n = 71)**	14 (19.72)	57 (80.28)	<1	8 (57.14)
			≥1	6 (48.86)
**Blood sugar test (n = 300)**	175 (58.33)	125 (41.67)	<1	98 (56.00)
			≥1	77 (44.00)
**Blood pressure test (n = 307)**	240 (78.18)	67 (21.82)	<1	129 (53.75)
			≥1	111 (46.25)
**Lipid profile (n = 181)**	68 (37.57)	113 (62.43)	<1	43 (63.24)
			≥1	25 (36.76)
**BMI evaluation (n = 152)**	87 (57.24)	65 (42.76)	<1	58 (66.67)
			≥1	29 (33.33)
**Waist circumference (n = 119)**	33 (27.73)	86 (72.27)	<1	22 (66.67)
			≥1	11 (33.33)
**Dental checkup (n = 309)**	132 (42.72)	177 (57.28)	<1	89 (67.42)
			≥1	43 (32.58)
**Eye checkup (n = 316)**	143 (45.25)	173 (54.75)	<1	90 (62.94)
			≥1	53 (37.06)
			**Number of times in the past 3 years**	

**Pap smear (n = 157)**	41 (26.11)	116 (73.89)	1	23 (56.10)
			2	12 (29.27)
			3	5 (12.20)
			>3	1 (2.43)
			**Number of doses**	

**HPV vaccination (n = 139)**	23 (16.55)	116 (83.45)	1	4 (17.39)
			2	5 (21.74)
			3	12 (52.17)
			4	2 (8.70)

### Reasons for the utilization and non-utilization of the preventive health services

For majority (60.65%) of the respondents, it was a personal decision to undergo these tests and 56.68% were influenced by a healthcare worker/provider. The least common influence was social media (10.83%). The most common reasons (62.22%) for not utilizing any of the preventive health services were the fact that they hadn’t heard about the tests before and the fact that they didn’t think it was important. The least common reasons (2.22%) were distance and not knowing where to do any of the tests. The cost of preventive healthcare services didn’t appear to be major reason for non-utilization in this as only 13.33% indicated cost as a reason.

### Factors affecting the knowledge and utilization of the preventive healthcare services

Socio-demographic characteristics of respondents did not significantly affect knowledge of the various preventive healthcare services (Table 6).

**Table 6:** Association between the respondents’ socio-demographic variables and knowledge of preventive healthcare services.

Variable	Knowledge of preventive health services			

	Poor (%)	Good (%)	Total (%)	X^2^ & P value

**Age (Years)**				

18–40	233 (89.73)	3 (1.27)	236 (100.00)	X^2^ = 1.10
41–65	86 (100.00)	0 (0.00)	86 (100.00)	Fisher’s P = 0.567
**Total**	319 (99.07)	3 (0.93)	322 (100.00)	
**Level of education**				

≤Secondary education	116 (100.00)	0 (0.00)	116 (100.00)	X^2^ = 1.71
Post-secondary	203 (98.54)	3 (1.46)	206 (100.00)	Fisher’s P = 0.556
**Total**	319 (99.07)	3 (0.93)	322(100.00)	
**Marital status**				

Not married	135 (98.54)	2 (1.46)	137 (100.00)	X^2^ = 0.72
Married/co-habiting	184 (99.46)	1 (0.54)	185 (100.00)	Fisher’s P = 0.577
**Total**	319 (99.07)	3 (0.93)	322 (100.00)	
**Employment status**				

Employed	201 (99.50)	1 (0.50)	202 (100.00)	X^2^ = 1.12
Unemployed	118 (98.33)	2 (1.67)	120 (100.00)	Fisher’s P = 0.558
**Total**	319 (99.07)	3 (0.93)	322 (100.00)	
**Estimated monthly income**				

≤18,000 Naira	123 (99.19)	1 (0.81)	124 (100.00)	X^2^ = 0.03
>18,000 Naira	196 (98.99	2 (1.01)	198 (100.00)	Fisher’s P = 1.000
**Total**	319 (99.07)	3 (0.93)	322 (100.00)	

Older women, women with post-secondary education, women who were married/co-habiting and women who earned more than the minimum monthly income had higher rates of undergoing the tests. Employment status didn’t seem to affect utilization of most of the preventive healthcare services.

## Discussion

The constant increase in the life expectancy and the rate of chronic diseases during the last decade has reinforced the need for health promotion, primary and secondary preventive services [19]. This study has shown that respondents were well aware of specified NCDs and preventive healthcare services and considered routine medical check-up important. However, they had poor knowledge of the preventive health services for NCDs and hardly utilized them. Awareness of specified preventive health services was generally good. This can be attributed to the increasing education of the public on these preventive health services by healthcare workers, use of multiple media services and interaction among family and colleagues.

Awareness of Mammogram though low (44%), is much higher than the 5% reported among female general out-patient clinic attendees in Ibadan, another Southwestern town. In that study, previous CBE, tertiary education and participation in community breast cancer prevention activities independently predicted this awareness [20].

It is quite surprising to note that a much lower proportion of the respondents were aware of Visual inspection tests (VIA/VILI) despite the fact that they are largely available at the Primary Health Care (PHC) level when compared to pap smear.

In another Lagos study, awareness of HPV Vaccine among mothers of adolescent girls was much lower (19.7%) [21] Overall, the respondents had better awareness rates than women from other studies in the northern, southern and southeastern parts of the country [18,22,23]. A number of reasons may account for this difference including the fact that Lagos is predominantly urban and the respondents had considerably high level of education when compared with respondents in the other studies.

Considering that the healthcare worker is a key source of information on preventive healthcare, interventions can expand their role in education efforts and sensitization of women at the community level to increase uptake of these preventive services.

Going a step further to determine their knowledge of these NCD preventive services and examinations, an abysmally poor knowledge was observed (using the summary measure). A very similar study in Uyo, southern Nigeria also showed poor knowledge [18]. The respondents were merely aware of the preventive healthcare services with inadequate knowledge. This knowledge gap has exposed the insufficiency of the information received by respondents. This inadvertently, may affect utilization.

Respondents in this study generally had poor utilization of NCD preventive healthcare services. They had better screening practices in terms of SBE, CBE, BP measurement, Blood sugar testing, BMI measurement, eye and dental checkup. Low utilization of preventive health services is not restricted to women in developing countries as African women in higher income countries were still found to utilize healthcare services less [24].

Screening practices such as mammogram, waist circumference (WC) measurement, lipid profile and pap smear were particularly low. This seems to be the case with female civil servants and cervical cancer screening, as only very few of them had ever screened for cervical cancer [22,25]. Civil servants are expected to have greater awareness, knowledge, and access to preventive healthcare services.

BP and BMI measurement are usually routinely done in health facilities and may account for the high figures although CBE rates compared well with US figures [26]. Better utilization rates were however expected for BP measurement and blood sugar testing as these are also readily available at the PHCs in addition to the annual state-wide hypertension and diabetes screening. This is very important because we do not have a national figure for prevalence of DM and most figures from pockets of studies are usually facility-based, hence under-reported. It is therefore vital that women should check their blood sugar levels at least once a year. Mammogram appears to be a luxury in low resource settings like Nigeria and other countries in Africa as even lower figures were reported by authors [12,27], quite in contrast with developed countries [28,29]. Despite the availability and cost-effectiveness at the PHC level, many women did not undergo VIA/VILI testing as much as pap smear test because they mostly attended secondary health facilities. VIA/VILI are usually done at the PHC level and costs between four to five US Dollars.

Results showed that individual factors such as older age, higher education and higher income/allowances (above the minimum monthly income) positively influenced screening for gynaecological cancers, similar to findings from other studies [18,27,30,31,32]. This underscores the importance of these individual factors in the prevention of cancers.

Just like in this study, women in southern Nigeria also had very low rates for lipid profile testing [18]. Cost may be the issue here as the test is rather expensive (about 14 US Dollars). Utilization of dental checkup was low, supporting previous observations that people hardly visit the dentist for routine checkup [33,34,35].

Slightly more than one in every ten respondents had not done any of the tests, higher than the proportion (6.5%) reported in southern Nigeria [18]. A combination of personal decision and influence of health worker were the main reasons for screening whereas in eastern Nigeria, community-based advocacy was a major motivator for cervical cancer screening [36]. Barriers to gynaecological cancer screening could be religious, cultural, ignorance about screening, fear of positive result, financial and even spiritual [27,37,38]. For the women who had never done any tests at all in this study, ignorance of the preventive services and poor attitude towards NCD screening were major barriers. In South Africa, even when preventive healthcare such as cancer screening, cholesterol testing, DM screening, eye and dental examination was incentivized, the utilization, though increased, was still sub-optimal [39], thus exposing how little or no importance is placed on NCDs by women.

Despite the magnitude of the NCD problem and the projections that it would get worse if actions are not taken, individuals have argued that irrespective of the apparent gains of periodic screening, the risks of side effects such as anxiety, overtreatment and over diagnosis are often ignored, and therefore recommend that we focus on problems that cause poor health *ab-initio* [40]. These draw backs potentially affect utilization.

### Strengths and limitations

There is a dearth of published data on this subject as authors have largely studied single diseases and their screening especially the gynaecological cancers. This study has covered the research gaps in addition to showing how insufficient the information about NCDs and their prevention is. Women have also been found to be very important in exacting change in the household and community at large and this will help in winning the war against NCDs [2].

It was conducted in a low-income country using robust sampling methods and covered a wide range of NCD preventive services, thereby generating much needed data on the awareness, specific knowledge and utilization of these services/tests. It also serves as an indirect evaluation of the state-wide screening programmes which have now been going on for about a decade.

The study was conducted in one Local Government Area of Lagos State, Nigeria and so results may not necessarily be generalized to the country. The study focused majorly on early detection of these NCDs. It reiterates the importance of primordial and primary prevention strategies but failed to study women’s knowledge of risk factors associated with NCDs and approaches to preventing these risk factors.

## Conclusions

This study demonstrated that although majority of the respondents were aware of the preventive healthcare services, knowledge and utilization of these NCD preventive healthcare services was very poor. With the healthcare worker being the major source of information, the accuracy of the information is assured and every opportunity to educate women on these NCDs and their prevention must be utilized.

Utilization of other mass media to deliver more specific messages on the functions, benefits and recommended frequency of the tests for NCD prevention and control should be explored for wider coverage.

Further larger scale research is still required probably using a combination of methods to study many facets currently not exploited with this study and also to elucidate barriers to utilization.
